# USER PERCEPTION OF TECHNOLOGICAL INTERVENTIONS FOR PARKINSON’S DISEASE: A SYSTEMATIC REVIEW

**DOI:** 10.1093/geroni/igad104.2451

**Published:** 2023-12-21

**Authors:** Terence Lau, Angela Y M Leung

**Affiliations:** The Hong Kong Polytechnic University, Hong Kong, Hong Kong; Hong Kong Polytechnic University, Hong Kong, Hong Kong

## Abstract

With the growing number of Parkinson’s disease technological intervention studies available, it is uncertain what and how people with Parkinson’s disease (PWP) respond to these interventions. This systematic review aims to study the user perception and perceived psychosocial gain of technological interventions among the Parkinson’s disease population, and it included 27 eligible studies from 6 databases. Through thematic synthesis, qualitative evidence suggests technological interventions could benefit the psychosocial well-being of people with Parkinson’s disease (PWP) under appropriate implementation. Coping with technological interventions was identified as the overarching theme. The theme is closely related to participants’ technological literacy and could lead to perceived psychosocial gain. The discrepancy between the implemented technology and PWP’s technological literacy level dictated how well PWP coped with the interventions. Ineffective coping attempts could induce undesirable emotions within PWP and tamper with their psychosocial health. In contrast, successful coping attempts reduced initial adverse effects, resulting in psychosocial growth such as increased autonomy and interpersonal relationships. To facilitate PWP’s coping process and design a Parkinson’s disease-friendly technological intervention. Scholars should include an adjustable, progressive module design and provide sufficient training and technical support to participants during implementation.

